# Evidence That Metapopulation Dynamics Maintain a Species' Range Limit

**DOI:** 10.1111/ele.70128

**Published:** 2025-05-06

**Authors:** Graydon J. Gillies, Michael P. Dungey, Christopher G. Eckert

**Affiliations:** ^1^ Department of Biology Queen's University Kingston Ontario Canada; ^2^ Department of Geography Memorial University of Newfoundland and Labrador St. John's Newfoundland and Labrador Canada

**Keywords:** biogeography, dispersal, habitat occupancy, landscape ecology, metapopulation, patch dynamics, plant ecology, range edge, range limit, species distributions

## Abstract

The metapopulation hypothesis for range limits proposes that geographic variation in a species' extinction from and/or colonisation of habitat can generate an abrupt range limit. We tested whether this contributes to the northern range limit of coastal dune plant *Camissoniopsis cheiranthifolia* by quantifying suitable habitat area and the rates of extinction and colonisation across 3485 plots throughout the northern half of the species' range. Colonisation of previously unoccupied plots increased with suitable habitat area and abundance in nearby plots and consequently declined towards the range limit. Extinction was more frequent from plots with less habitat and lower initial abundance but did not increase significantly towards the limit. Incorporating spatial variation in estimated rates of colonisation and extinction in a metapopulation model predicted a decline in plot occupancy towards the limit that closely matched the observed decline in occupancy. Thus, variation in metapopulation dynamics may contribute to this species' range limit.

## Graphical abstract

The metapopulation hypothesis for species range limits posits that a range limit may be imposed by subtle variation in rates of either patch colonisation or extinction. Using a multi‐year survey across ~900 km of the Pacific coast of the USA, we found evidence for key predictions made by the metapopulation hypothesis using coastal dune endemic *Camissoniopsis cheiranthifolia* , including a decline in colonisation rates and patch occupancy towards the northern range limit.

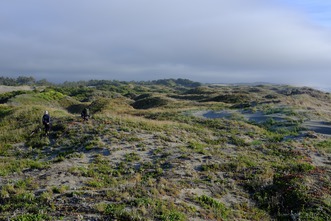

## 1 Introduction

Limits to species' distributions are thought to arise because species are unable to persist in or disperse to suitable habitat beyond their range (Gaston 2009). In an era of rapid environmental change caused by humans, it is expected that many species will shift their ranges to track suitable climates (Burrows et al. 2014; Chen et al. 2011; Davis and Shaw 2001; Lenoir and Svenning 2015). Understanding the processes that maintain species' range limits and predicting whether species will overcome obstacles to range expansion is not only a fundamental ecological question but also valuable for designing forward‐facing conservation programs. Yet, mechanisms that explain the maintenance of range limits remain inadequately tested.

That geographic range limits are caused by low fitness in habitats beyond the range (i.e., niche limitation) is supported in some species (Cross and Eckert 2020), but experimental populations transplanted beyond geographical range limits often realise fitness adequate for self‐replacement (Hargreaves et al. 2014). In these cases, the range limit is explained by invoking some form of dispersal‐limitation without this being tested directly. The simplest possibility is that the species' dispersal is inadequate to colonise suitable habitat beyond the limit, either because that habitat is too rare, or dispersal is impeded. A more nuanced alternative to simple niche vs. dispersal limitation, inspired by metapopulation theory, involves geographic variation in habitat patch dynamics. Assuming habitat is patchily distributed, as is generally the case (Husband and Barrett 1996; Watling et al. 2011), some proportion, *n*, of habitat patches are, at any given time, occupied (*n* ≤ 1), and the level of occupancy is determined by the balance between the rate at which the species becomes extinct from occupied patches (*e*) versus the rate at which vacant patches of suitable habitat are colonised (*c*). If a species can colonise suitable but vacant patches at least as quickly as it goes extinct from occupied patches (*c* ≥ *e*), then the metapopulation is maintained (*n* > 0). However, if colonisation declines or extinction increases (*c* < *e*), then the metapopulation may collapse (*n* = 0) (Hanski 1998; Levins 1969). Theory confirms that metapopulation dynamics could enforce range limits through geographical gradients in colonisation (*c*) and/or extinction (*e*), and that abrupt range limits can arise from subtle changes in just one or both parameters (Carter and Prince 1981; Holt and Keitt 2000; Lennon et al. 1997). While the prevalence of metapopulations, as envisioned by theory, has been debated (Fronhofer et al. 2012), the metapopulation framework can provide a useful context for better understanding how population dynamics change towards species' range limits where habitat availability and/or niche breadth declines (Oliver et al. 2009; Thomas et al. 1999).

Both niche and dispersal limitation are subsumed in the metapopulation hypothesis. Extinction may increase if habitat quality and hence fitness decline towards the range limit. However, extinction can also increase with stochastic disturbance independent of variation in fitness (Wilcox et al. 2006). Similarly, colonisation may vary with the species' capacity for movement and successful dispersal to suitable habitat (Ebenhard 1991; Johst et al. 2002), local propagule pressure (Hufbauer et al. 2013), the spatial distribution of vacant but suitable habitat (i.e., habitat availability; Xu et al. 2006), and the success of the species in moving through the habitat between suitable patches (‘matrix permeability’; Vandermeer and Carvajal 2001). Dispersal limitation can, therefore, be more nuanced than a species simply not being able to access suitable habitat beyond its range. The geographical range limits of many species appear to not be imposed solely by niche or dispersal limitation (Hargreaves et al. 2014), and thus the metapopulation hypothesis for range limits addresses a long‐standing conundrum by integrating both processes under one framework. Despite this, the metapopulation hypothesis has not to our knowledge been directly tested because rates of colonisation and extinction have not been measured in natural habitat towards a species' geographic range limit.

Here, we conducted a large‐scale cross‐generation survey of habitat availability and occupancy to test the prediction that changes in patch colonisation and/or extinction rates might reduce occupancy towards the northern range limit of the Pacific coastal dune plant *Camissoniopsis cheiranthifolia* , a short‐lived perennial (Samis et al. 2016). Transplants of *C. cheiranthifolia* have shown that the species is capable of persisting at least 220 km beyond its northern range limit with high fitness, suggesting niche limitation alone is not responsible for maintaining this range limit (Cross and Eckert 2021). However, simple dispersal limitation is also unlikely, as coastal dune habitat seems to be widely available beyond the range limit according to surveys of plant community composition (Samis and Eckert 2009) and the long‐term survival of beyond‐range transplants (Cross and Eckert 2021). In coastal dunes, suitable habitat for foredune species like *C. cheiranthifolia* is highly fragmented by dense, relatively undisturbed vegetation or highly disturbed open sand. Populations in suitable habitat likely experience frequent stochastic extinction (Brunbjerg et al. 2012; McLachlan 1991), a process often missed by short‐term transplant experiments. Data from a preliminary survey showing declining patch occupancy by *C. cheiranthifolia* were consistent with the metapopulation hypothesis (Samis and Eckert 2009), but sample sizes were small and there was no temporal replication to estimate colonisation and extinction. As such, the metapopulation hypothesis has not yet been formally tested. Here, we collected occupancy and habitat suitability data from 3485 spatially randomised plots in coastal dune habitat across two generations along 938 km of coastline from San Francisco, California, USA to the species' northern range limit near Dunes City, Oregon, USA. We test the following predictions to investigate the metapopulation hypothesis for range limits:

1. Colonisation is more frequent in vacant plots that contain more suitable habitat and have a greater local abundance of *C. cheiranthifolia* in neighbouring plots (i.e., are hypothesized to have greater propagule pressure).
2. The probability that vacant but suitable plots are colonised declines towards the range limit. We expect declining local abundance and suitable habitat per plot towards the range limit to cause the expected decline in colonisation.
3. Extinction is more frequent from plots that have a lower initial abundance because smaller populations should be more prone to stochastic extinction (Lande 1993), have less suitable habitat because populations residing in smaller habitat patches tend to be more prone to extinction (Fahrig 1997; MacArthur and Wilson 1967), and have lower abundance in nearby plots (lower propagule pressure increases extinction risk; Brown and Kodric‐Brown 1977; Eriksson et al. 2014).
4. The probability that the species goes extinct from plots increases towards the range limit. We expect declining initial plot abundance and suitable habitat per plot towards the range limit to cause the expected increase in extinction.
5. Plot occupancy declines towards the range limit. If the range limit is stable and changes in occupancy are caused by variation in colonisation and/or extinction, we expect the decline in actual plot occupancy to closely match the predicted equilibrium occupancy calculated using metapopulation models from measured rates of colonisation and extinction.

Our findings suggest that geographic variation in metapopulation dynamics, particularly a decline in colonisation, is sufficient to cause a decline in occupancy towards the range limit that might contribute to metapopulation collapse and thus the generation of this species' abrupt northern range limit.

## 2 Methods

### 2.1 Study System

*Camissoniopsis cheiranthifolia* (Hornem. Ex Spreng.) W.L. Wagner & Hoch (Onagraceae) is a small, herbaceous, short‐lived perennial plant endemic to the Pacific coastal dunes from Baja California, Mexico to southern Oregon, USA (Figure S1; López‐Villalobos and Eckert 2019). This species is excellent for testing the metapopulation hypothesis. First, beyond‐range transplants strongly suggest that the northern range of the species is not entirely niche‐limited because experimental populations have enjoyed high fitness for many generations even 220 km north of the limit (Cross and Eckert 2021, 2024). Its small seeds are readily dispersed via wind or water, and dune habitat exists abundantly across the northern range limit (Samis and Eckert 2009), making it unlikely that the species simply cannot disperse to habitat beyond the range. Moreover, this species seems to have undergone long‐distance dispersal in the past, as its current geographical range transcends large regions of rocky coastline such as the Lost Coast and Big Sur, CA, where no dune habitat exists. It has also colonised the Channel Islands off southern California (GBIF.org 2024). Thus, the mechanisms maintaining the northern range limit in this species remain unclear. Third, coastal dune habitat is ‘patchy’, with patches of suitable habitat separated by open disturbed sand, dense vegetation (Pickart 2013), and bodies of water, suggesting that metapopulation dynamics could influence the species' distribution. Fourth, the species' short generation time of 1.0–1.9 years (Samis et al. 2016) in conjunction with frequent habitat disturbance (Brunbjerg et al. 2012; Johnson and Miyanishi 2010; Miller 2015) means that *C. cheiranthifolia* can newly colonise or go extinct from habitat patches rapidly. Finally, very high germination rates under greenhouse conditions (Cross 2022) suggest that its small seeds do not have prolonged dormancy such that the species is unlikely to have a deep seed bank. Hence, we can reliably assess habitat as being occupied or not.

### 2.2 Multi‐Year Survey

We generated 5418 random coordinates within putative coastal dune habitat across the northern half of *C. cheiranthifolia* 's distribution (Supporting Information S2). In summer 2019, we visited each point that was accessible and, in 2022, revisited all points that were accessible and confirmed to be in coastal dune habitat. Points were located and relocated using a Garmin GPSmap 60Cx, with precautions taken to ensure GPS‐induced relocation error was minimal (Supporting Information S3). At each point, we established 5 × 5 m plots centred on the point and parallel to the coastline. The northern range limit, occurring at 43.8° N (Figure S1), is also indicated by recent geographical surveys and herbarium records collected over the last century (Samis and Eckert 2007). We indexed patch size by estimating the total area of suitable habitat (0–25 m^2^) within each plot following Samis and Eckert (2007), hereafter ‘suitable plot area’. Confidence in our ability to identify suitable habitat comes from multiple range‐wide surveys (Samis and Eckert 2007, 2009) as well as the high fitness of experimental populations transplanted into what we classified as suitable habitat (Cross and Eckert 2021). Our index for suitable plot area will underestimate patch sizes for patches larger than 5 × 5 m but provides a measure of the availability of habitat directly at the plot locations. We also counted *C. cheiranthifolia* abundance in plots occupied by the species, and for each plot containing any amount of unsuitable habitat, recorded the reasons for unsuitability.

### 2.3 Assessing Metapopulation Structure and Dynamics

All analyses were conducted in R (v. 4.3.2; R Core Team 2023) and all models were fit using the *glmmTMB* function in the glmmTMB package (v. 1.1.8; Brooks et al. 2017). We excluded all plots that turned out to not include coastal dune habitat (i.e., consisting entirely of water, pavement, or beach), leaving *n*_*2019*_ = 3485 and *n*_*2022*_ = 3289 coastal dune plots. For binary analyses, plots were classified as suitable if they contained some suitable habitat. We used the *Anova* function in the car package (v. 3.1–2; Fox and Weisberg 2019) to perform type II significance tests for all predictors.

We defined colonisation, *c*, as the transition from unoccupied (no *C. cheiranthifolia* ) in 2019 to occupied (≥ 1 individual) in 2022, for all plots that had the potential to be colonised (suitable in 2022, but not necessarily suitable in 2019). We defined extinction, *e*, as the transition from occupied to unoccupied using all plots that were occupied in 2019 (regardless of suitability in 2022). Treating the coastline as a continuous metapopulation system, we modelled spatial variation in both *c* and *e* using binomial generalised linear models (GLMs) with distance to the range limit as the sole predictor.

We assessed whether metrics of habitat structure and the spatial distribution of *C. cheiranthifolia* caused variation in *c* and *e*. To assess which covariates were associated with greater likelihoods of plot colonisation, we modelled variation in *c* as a binary response variable with suitable plot area (the area of the plot that contains suitable habitat, m^2^), local abundance of *C. cheiranthifolia* in 2019 (the average number of *C. cheiranthifolia* in all other plots within 500 m of the focal plot, chosen because dispersal has been recorded at this spatial scale after ~10 generations; Cross and Eckert 2021), and their interaction as predictors. We modelled variation in *e*, again as a binary response, with the abundance of *C. cheiranthifolia* in the focal plot during 2019, 2019 local abundance, 2019 suitable plot area, and the interaction between plot abundance and local abundance as predictors. To account for spatial autocorrelation, we used the coordinates of each plot in a covariance structure that assumes an exponential decay in correlation with distance in the models (Kristensen and McGillycuddy 2024) and compared the AICc of models with and without the covariance structure. For both models, including the covariance structure yielded a lower AICc (colonisation model: 956.16 with, 1032.78 without; extinction model: 602.92 with, 632.07 without), so the term was kept in the models.

To test for spatial variation in the prevalence of extinctions caused by demographic vs. environmental stochasticity, we tested whether plots from which the species had gone extinct remained suitable (demographic extinctions) or became unsuitable (environmental extinction). To test whether the prevalence of the two major environmental causes of extinction varied towards the range limit, we modelled spatial variation in the proportion of plots where *C. cheiranthifolia* went extinct due to successional extinction (change in vegetation) vs. disturbance extinction (transition to wind‐blown, disturbed sand), each as a binary variable with distance to the range limit as the sole predictor. We expect that, towards the northern range limit, environmental extinctions (specifically by wind disturbance) become more common due to the greater intensity of storms at higher latitudes on the Pacific coast (Bromirski et al. 2017). Furthermore, we evaluated whether the variables that possibly influence colonisation and extinction (suitable plot area in 2019 and 2022, log_10_ initial plot abundance, and log_10_ local abundance in 2019) vary towards the range limit by fitting each to a Gaussian GLM with distance to the range limit as the sole predictor.

The metapopulation hypothesis predicts that a range limit occurs at the point where the rates of colonisation and extinction are such that the species no longer occupies patches of suitable habitat. Theoretically, equilibrium plot occupancy (n*) in a metapopulation (i.e., the steady‐state proportion of plots occupied assuming constant colonisation and extinction) can be predicted from rates of colonisation (*c*) and extinction (*e*) (Hanski 1994):

(1) $$n^{*}=\frac{c}{c+e}$$

Thus, n* will vary geographically in response to changes in either (or both) parameters *c* and *e*. We used this metapopulation model (Equation 1) to test whether variation in *c* and *e* towards the range limit is sufficient to account for geographic variation in patch occupancy (*n*_obs_). First, we modelled variation in observed patch occupancy of suitable plots (*n*_obs_) as a binary variable using a binomial GLM with distance to the range limit as a predictor. We then used the *predict* function to extract expected values from the models of *c* and *e* towards the range limit (above) and used these predicted values to calculate equilibrium occupancy predicted by Equation (1) (n*) at each point across space. This generated a pattern of variation in n* towards the range limit that could be attributed to variation in *c* and *e* alone. To generate a confidence envelope around this regression, we re‐ran the colonisation and extinction models on 1000 bootstraps of the data and estimated the regression of n* over distance to the range limit for each bootstrap. Similarity between the geographic patterns of *n*_obs_ and n* is not an inevitable outcome of both being estimated from the same data because *n*_obs_, *c*, and *e* were each calculated from a different subset of the data as described by Holt and Keitt (2000). Moreover, *c* and *e* were estimated from changes in occupancy between both survey years and are thus mostly independent from the occupancy status of plots in 2022.

## 3 Results

### 3.1 Colonisation Declines Towards the Range Limit

Overall, 19.0% of suitable but vacant plots were colonised by *C. cheiranthifolia* (*c* = 0.190) and the species went extinct from 30.6% of occupied plots (*e* = 0.306). The proportion of suitable plots that were colonised was substantially lower than the proportion of occupied plots that went extinct, but because the pool of plots from which we calculated colonisation (1223 plots) was much greater than the pool from which we calculated extinction (638 plots), the actual number of observed colonisation and extinction events was similar (232 vs. 195, respectively). Across all plots, occupancy was 22.2% in 2019 and 22.4% in 2022.

**FIGURE 1:**
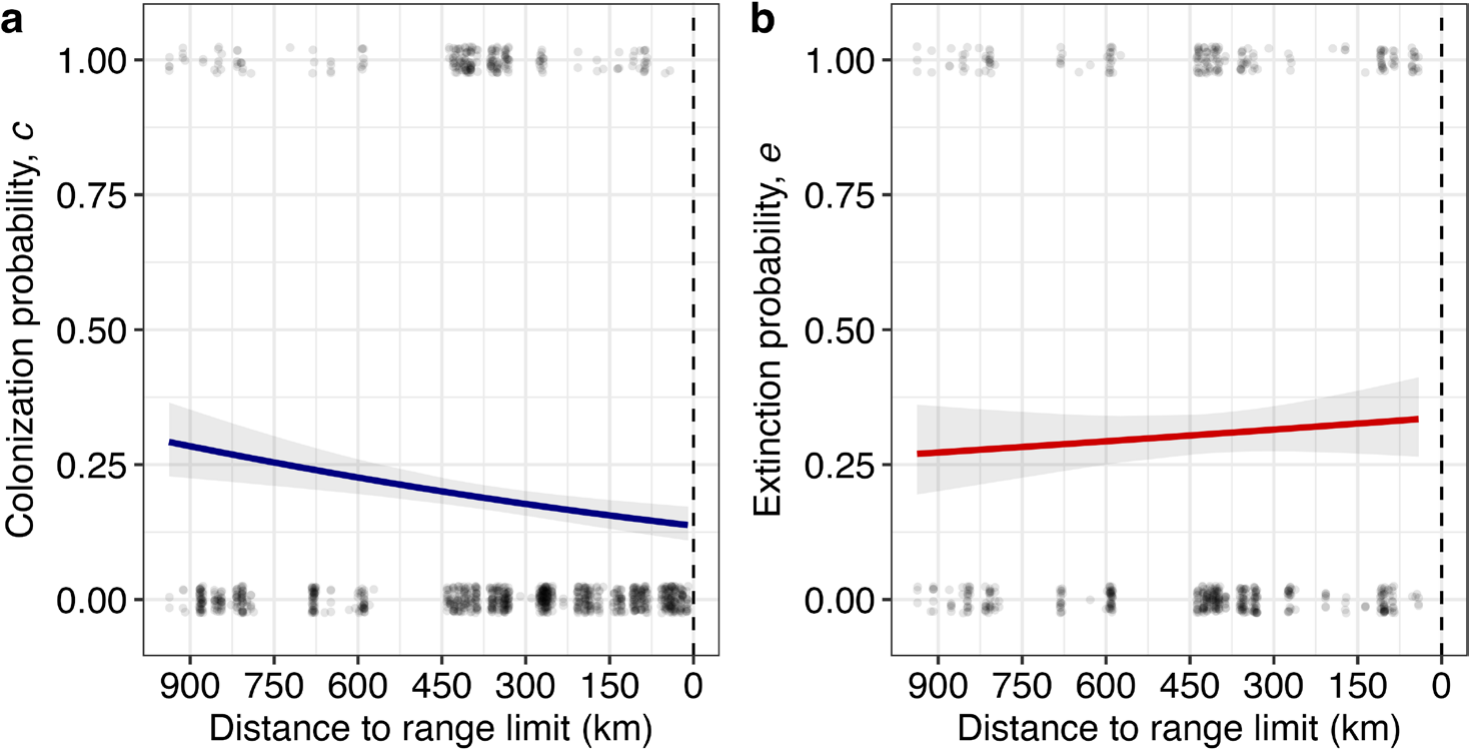
(a) Colonisation (*c*) declines significantly towards the northern range limit of *Camissoniopsis cheiranthifolia* while (b) extinction (*e*) increases but not significantly. Dashed vertical lines indicate the species' northern range limit. Grey ribbons indicate 95% confidence envelopes. Points are jittered from 0 and 1 in the y‐dimension and the *x*‐axis scale is reversed for clarity.

Colonisation declined significantly towards the range limit (*n* = 1223, *b* = 1.02 × 10^−3^ log‐odds, *χ* ^2^ = 13.46, df = 1, *p* < 0.001; positive coefficient indicates an increase towards the southern range core; Figure 1a), while extinction increased slightly but not significantly (*n* = 638, *b* = −3.41 × 10^−4^ log‐odds, *χ* ^2^ = 0.83, df = 1, *p* = 0.36; Figure 1b). Of the 195 plots where *C. cheiranthifolia* went extinct between surveys, 71.4% still included suitable habitat in 2022. However, fitting a binomial GLM of habitat suitability (binary suitable or unsuitable) to distance to the range limit revealed that distance did not predict the likelihood that a plot remained suitable after extinction (*n* = 189, *b* = −1.07 × 10^−5^ log‐odds, *χ* ^2^ = 3.0 × 10^−4^, df = 1, *p* = 0.99, using all plots experiencing extinction excluding six that were missing data on the cause of unsuitability). Of the 54 of 189 plots where extinction was associated with the habitat becoming unsuitable, 57.4% became unsuitable due to increased vegetation cover (hereafter ‘successional extinction’), 38.9% due to wind disturbance, 5.6% were covered by driftwood, and 1.9% were covered by water (percentages add up to > 100 because a single plot may become unsuitable via multiple processes). Successional extinction increased and wind disturbance extinction declined towards the range limit but both trends were not quite significant (*p* = 0.097 and *p* = 0.061, respectively; Figure S2).

### 3.2 Patch and Population Parameters Predict Colonisation and Extinction

**FIGURE 2:**
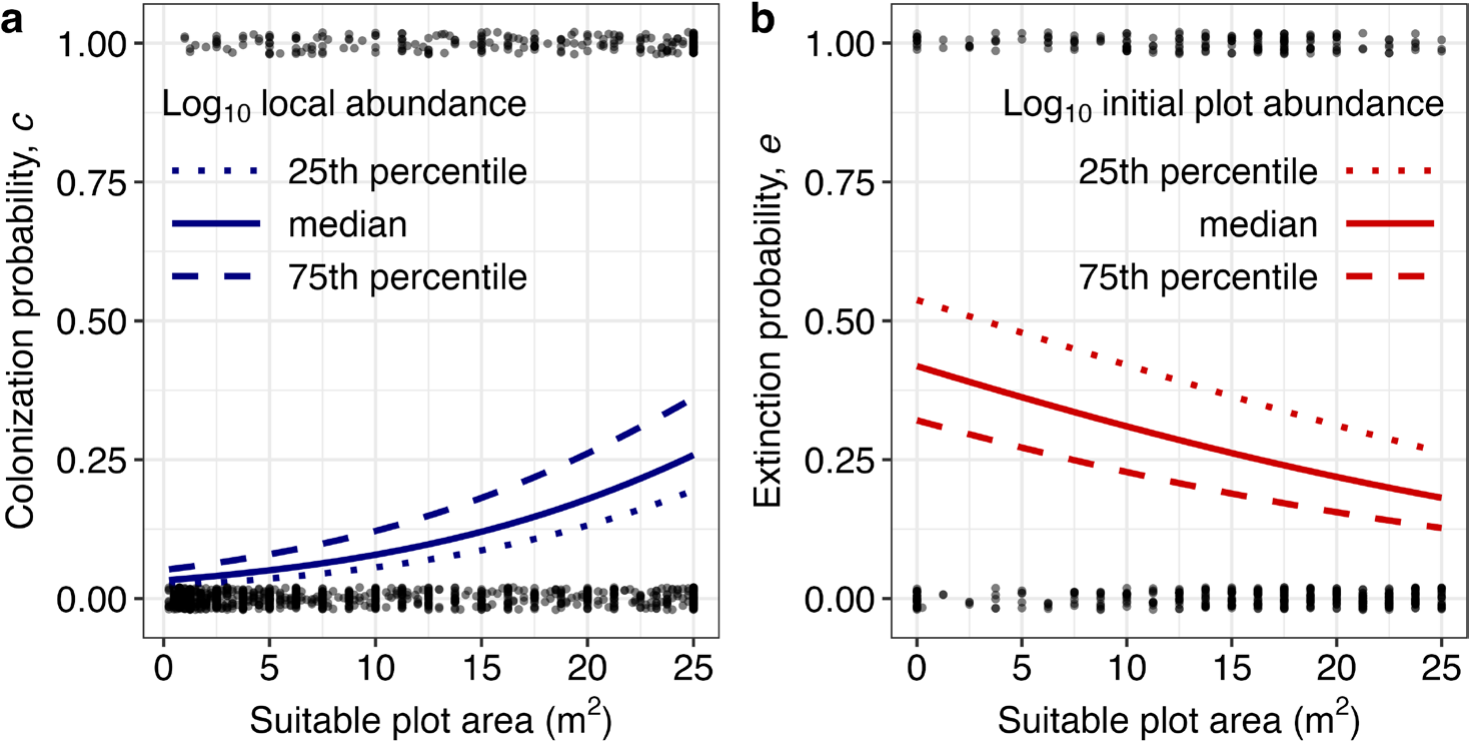
(a) Colonisation of vacant plots (*c*) by *Camissoniopsis cheiranthifolia* increases with suitable plot area and local abundance, while (b) the rate of extinction from occupied plots (*e*) declines with suitable plot area and initial plot abundance. The colonisation model includes all plots that could be colonised (vacant in 2019 and containing suitable habitat in 2022) and had complete data for local abundance and suitable plot area, while the extinction model includes all plots that could have gone extinct (contained *C. cheiranthifolia* in 2019) and had complete data for suitable plot area and initial plot abundance. In panel (a), the dotted, solid, and dashed lines indicate predicted colonisation rates when the log_10_‐transformed local abundance (the average *C. cheiranthifolia* abundance from all neighbouring plots within 500 m) is held constant at the 25th, 50th, and 75th percentiles, respectively. In panel (b), the same lines indicate predicted extinction rates when log_10_‐transformed initial plot abundance is held constant at those percentiles with local abundance (not significant) held constant at its median. Points are jittered from 0 to 1 in the y‐dimension for clarity.

As expected, colonisation was more likely at plots containing more suitable habitat (*n* = 1221, *b* = 0.093 log‐odds, *χ* ^2^ = 53.58, df = 1, *p* < 0.0001) and with higher local abundance of *C. cheiranthifolia* (*b* = 0.73 log‐odds, *χ* ^2^ = 12.23, df = 1, *p* = 0.00047) but we did not detect a significant interaction (*b* = 0.00035 log‐odds, *χ* ^2^ = 0.0003, df = 1, *p* = 0.99; Figure 2a). Both suitable habitat area and local abundance declined significantly towards the range limit (Figure S3). Extinction was more likely at plots with lower initial abundance of *C. cheiranthifolia* (*n* = 538, *b* = −0.81 log‐odds, *χ* ^2^ = 20.25, df = 1, *p* < 0.0001) and less suitable plot area (*b* = −0.047 log‐odds, *χ* ^2^ = 6.05, df = 1, *p* = 0.014; Figure 2b) but we did not detect an interaction (*b* = −0.18 log‐odds, *χ* ^2^ = 0.21, df = 1, *p* = 0.65) or an effect of local abundance (*b* = −0.20 log‐odds, *χ* ^2^ = 2.37, df = 1, *p* = 0.12). Only initial plot abundance in 2019 declined towards the range limit (Figure S3). As a post hoc analysis, we included the predictive patch and population covariates in the colonisation model with only distance to the range limit as a predictor and found the effect of distance was no longer significant and the regression coefficient declined in magnitude (Table S1).

### 3.3 Metapopulation Occupancy Structure

**FIGURE 3:**
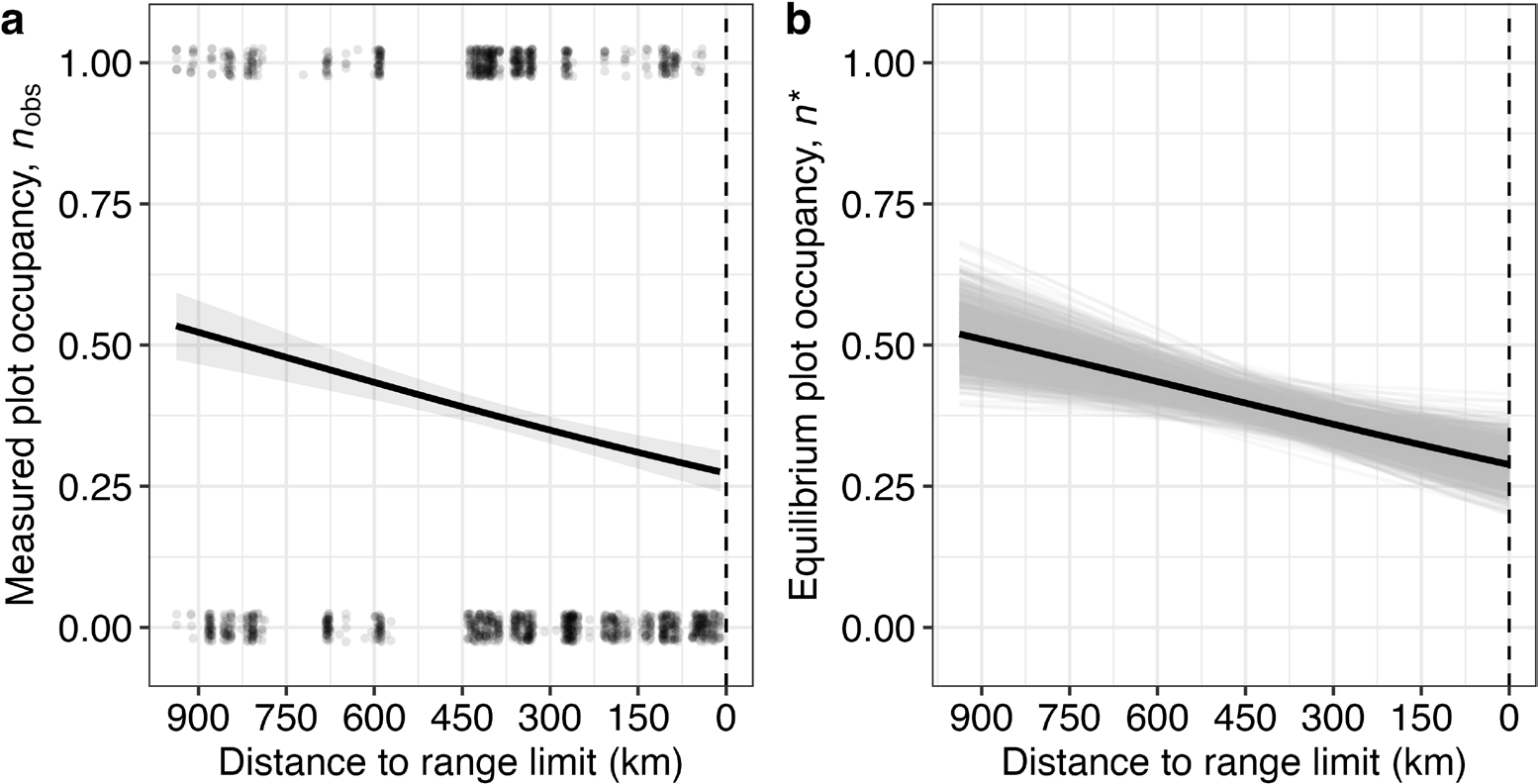
(a) The observed occupancy of suitable plots (*n*_obs_) in 2022 declines towards *Camissoniopsis cheiranthifolia* 's northern range limit, as does (b) the predicted equilibrium occupancy of plots (n*) calculated from estimated rates of colonisation (*c*) and extinction (*e*). The grey ribbon in (a) represents the 95% confidence envelope. Notably, the predicted equilibrium line (n*) falls entirely within the confidence intervals of the measured occupancy (*n*_*obs*_) model (not shown). The grey lines in panel (b) denote a confidence envelope generated from re‐running the calculation on 1000 bootstraps of the data. The vertical dashed line indicates the species' northern range limit. The occupancy model is fit using all patches within the range limit that contain suitable habitat. Points in (a) are jittered from 0 and 1 in the y‐dimension, and the x‐axis scale is reversed for clarity.

Observed plot occupancy of suitable plots (*n*_obs_) declined dramatically towards the range limit, from 0.534 at the southern end of the survey to 0.273 at the limit (*n* = 1802, *b* = 1.19 × 10^−4^ log‐odds, *χ* ^2^ = 34.79, df = 1, *p* < 0.0001; Figure 3a). Observed occupancy of suitable plots in 2019 also declined (*n* = 1669, *b* = 1.21 × 10^−3^ log‐odds, *χ* ^2^ = 39.15, df = 1, *p* < 0.0001; not shown). Using predicted values of *c* and *e* across the range (Figure 1), Equation (1) predicted a decline in equilibrium plot occupancy (n*) that almost perfectly matched the geographical trend in observed occupancy (*n*_obs_) (0.520 at the range core to 0.288 at the limit; Figure 3b). Furthermore, the geographic pattern of n* fell entirely within the 95% confidence interval around the pattern for *n*_obs_ (Figure 3). The close match between observed and predicted patch occupancy was not an inevitable consequence of both being estimated from the same survey data, as the rates of *c*, *e* and *n*_obs_ are each calculated from separate subsets of the data, and thus the mathematical model used to predict n* is independent of the statistical model used to test for variation in *n*_obs_.

## 4 Discussion

The potential importance of metapopulation dynamics for species occurrence across landscapes is well understood (Carter and Prince 1981; Holt and Keitt 2000; Lennon et al. 1997), but to our knowledge these dynamics have not yet been explicitly measured towards geographic range limits until now. The metapopulation hypothesis predicts that a range limit can arise from subtle changes in extinction and/or colonisation. As predicted, we found geographic variation in colonisation and, to a lesser extent, extinction (Figure 1). Further, the joint pattern of geographic variation in estimated *c* and *e* predicted a northward decline in equilibrium occupancy (n*) that closely matched the decline in observed plot occupancy (*n*_obs_) (Figure 3). That the geographic pattern of occupancy can be predicted by an equation that assumes equilibrium rates of *c* and *e* suggests that the combined effect of geographic variation in *c* and *e* adequately explains the dwindling occupancy of *C. cheiranthifolia* towards the range limit, and further suggests that the range limit may be stable, which is consistent with repeated surveys of this species across the northern portion of its range over a 20‐year period as well as analyses of a 150‐year history of herbarium records suggesting a stable limit over historical times (Samis and Eckert 2007).

We detected a colonisation rate of 19.0% within the range over three years (one generation), which is comparable to other estimates from plant metapopulations (Dornier et al. 2011; Jäkäläniemi et al. 2005). As expected, colonisation of vacant habitat patches by *C. cheiranthifolia* was predicted by suitable plot area and local abundance in nearby plots (Figure 3a). Both these covariates declined towards the species' northern range limit (Figure S3). Our post hoc analysis that found the effect of distance was reduced and non‐significant when these covariates were included in the model (Table S1) suggests that the decline in colonisation is primarily due to these changes in habitat structure and local abundance.

Extinction was prevalent, with *C. cheiranthifolia* going extinct from 30.6% of occupied plots (which is higher than some other recorded rates of patch extinctions; Bengtsson 1989; Dornier et al. 2011; Jäkäläniemi et al. 2005) (Figure 1b), possibly reflecting the rapid rate of change in coastal dune habitat. Extinction from a plot was predicted by lower plot abundance and suitable plot area in 2019 (Figure 2b). Although plot abundance declined towards *C. cheiranthifolia* 's range limit (Figure S3) we did not detect a corresponding increase in extinction towards the range limit (Figure 1b). However, including initial plot abundance and suitable plot area in the distance model reduced the magnitude of the distance coefficient (Table S1), suggesting these predictors may be partly responsible for explaining spatial variation in extinction. On one hand, these results are consistent with the results of transplant experiments that show *C. cheiranthifolia* enjoying high fitness towards and up to 220 km beyond the range (Cross and Eckert 2024; Samis et al. 2016; Samis and Eckert 2009) and with data showing that experimental populations can persist beyond the range for at least 10 generations (Cross and Eckert 2021). On the other hand, it is unclear empirically how geographic variation in stochastic extinction may play a role. Most plots from which *C. cheiranthifolia* went extinct still included some suitable habitat (71.4%), possibly suggesting a stronger role of demographic stochasticity than stochastic disturbance. We did not detect systematic variation in the odds that plots went extinct due to demographic vs. environmental change.

Of the plots where *C. cheiranthifolia* went extinct due to habitat change, 57.4% became unsuitable due to increased vegetation cover, suggesting succession (i.e., competition) may cause more extinction than wind disturbance (38.9%) on short timescales, although both were prevalent sources of extinction. Towards the range limit, there was an increase in successional extinction and a decline in wind disturbance extinction, though both trends were not quite significant (Figure S2). We predicted an increase in wind disturbance towards the northern range limit because Pacific storm surges that greatly disturb coastal dunes (Allan et al. 2011; Vellinga 1982) increase with latitude along the Pacific coast of North America (Bromirski et al. 2017). Thus, the processes that make habitat unsuitable and cause extinction of *C. cheiranthifolia* may vary across space, but the evidence is not conclusive.

Importantly, the decline in plot occupancy towards the northern range limit, both as observed and calculated (Figure 3), appears to be accounted for by geographic variation in plot colonisation and extinction rates. In particular, the close match between our calculated estimates of equilibrium occupancy n* across space with measured plot occupancy *n*_obs_ strongly suggests that patch dynamics are responsible for the dwindling occupancy. However, the question remains whether this decline is adequate to cause a range limit. For infinite metapopulations often used in theory, metapopulations may persist at infinitesimally small occupancy levels (see Ovaskainen and Hanski 2003). In reality, finite metapopulations exhibit an unstable equilibrium at low occupancy (i.e., Allee effects; Hanski and Gyllenberg 1993; Lande et al. 1998). Although occupancy of *C. cheiranthifolia* does not decline to zero, theory suggests that there is likely to be a non‐zero threshold for occupancy below which the metapopulation collapses (Hanski et al. 1996). First, there is likely feedback between occupancy and colonisation. Because colonisation of dune habitat patches decreases as the local abundance of *C. cheiranthifolia* declines, lower occupancy might necessarily reduce the rate of colonisation. Second, there is likely some stochasticity in metapopulation dynamics. In the same way that small populations are more prone to stochastic extinction (Gabriel and Bürger 1992; Matthies et al. 2004), sparse metapopulations are prone to collapse if there is stochasticity in colonisation and extinction rates (which is not accounted for in many metapopulation models; Carter and Prince 1981; Holt and Keitt 2000). It is possible that *C. cheiranthifolia* has reached its minimum viable patch occupancy (~27.3%) at its northern range limit, an occupancy rate that is similar to or exceeded by other estimates of minimum viable metapopulation size (Biedermann 2000; Hanski et al. 1996) and that the metapopulation collapses when occupancy is below this threshold. Confirming this threshold experimentally might require transplanting many subpopulations into habitat patches just beyond the range to then measure probabilities of *c* and *e* as well as long‐term changes in patch occupancy, an ambitious approach that has yet to be attempted. The hypothesis could be generally tested on a more practical scale by varying patch occupancy and tracking metapopulation fate in experimental ‘micro‐landscapes’ (Larsen and Hargreaves 2020).

What maintains species' geographic range limits has long been a central ecological question that has been brought into sharper focus by a general concern that species may not be able to shift their ranges rapidly enough to keep up with changing climates (Burrows et al. 2014; Talluto et al. 2017). Most recent work has tested the importance of niche limitation with very little attention on spatial processes (Cross and Eckert 2020) and no formal tests of the metapopulation hypothesis. We have shown, for the first time, that metapopulation dynamics, especially patch colonisation, vary geographically in a way that might contribute to maintaining the northern range limit of *C. cheiranthifolia* . This supports the long‐standing but untested theory that geographic variation in these processes can act to impose species' range limits and sets up a framework with which to evaluate the causes of range limits that goes beyond the dichotomy of niche versus dispersal limitation. There is still much empirical work on the topic that is yet to be done, such as understanding how range limits may emerge from interspecific competition within and across patches (Case et al. 2005). Furthermore, work on range limits has overwhelmingly focused on understanding how individual fitness and population demography change towards range limits in ways that might cause extinction beyond the range limit (Cross and Eckert 2021; Hargreaves et al. 2014; Sexton et al. 2009), but our results suggest that changes in colonisation might be at least as or more important than extinction.

Beginning to recognise the importance of potentially subtle changes in colonisation and extinction dynamics and the spatial distribution of appropriate habitat towards and at range limits may help us better predict the capacity of species for range expansion in response to rapid environmental change and lead to more effective conservation action. Anthropogenic habitat fragmentation and degradation may increase extinction but may also impede the movement of species between remaining patches of suitable habitat. While the importance of habitat connectivity for maintaining populations of motile animals has long been recognised (Correa Ayram et al. 2016; Watling et al. 2011), it is rarely discussed for conservation of sessile organisms like plants. Our study also demonstrates the potential challenges of conserving range‐edge populations, which are especially important for species that reach their range limits and are at risk just within the equatorial borders of high latitude jurisdictions, such as Canada (Caissy et al. 2020) or Finland (Komonen 2007), where human activity is most intense (Coristine and Kerr 2011). Consequently, this greater intensity of human activity may reduce colonisation and increase extinction towards range limits where habitat structure may already be increasingly unfavourable for stable metapopulation dynamics. Thus, peripheral populations may be particularly sensitive to further habitat fragmentation and require targeted conservation. Adding to this the requirement that species may need to undergo climate‐driven range shifts (Chen et al. 2011; Davis and Shaw 2001) strongly emphasises the importance of conserving habitat continuity.

## Author Contributions

Michael P. Dungey and Christopher G. Eckert conceived and designed the study. All authors collected data. Graydon J. Gillies analysed the data and wrote the manuscript with critical contributions from Christopher G. Eckert. All authors gave final approval for publication.

### Peer Review

The peer review history for this article is available at https://www.webofscience.com/api/gateway/wos/peer‐review/10.1111/ele.70128.

## Supporting information


Data S1.

## Data Availability

Data and code available from Zenodo digital repository: https://doi.org/10.5281/zenodo.14860282 (Gillies et al. 2025).
