# Supplementary material for: Evidence That Metapopulation Dynamics Maintain a Species' Range Limit
Source: Ecol Lett. 2025 May 6;28(5):e70128. doi: 10.1111/ele.70128 (PMC12053793; doi:10.1111/ele.70128)
Supplement: Supplementary file 1 — Data S1. [file ELE-28-0-s001.docx]

**Supporting Information for: Evidence that metapopulation dynamics maintain a species’ range limit**

**G. J. Gillies, M. P. Dungey, & C. G. Eckert**

**Supporting Information S1 – Figures and Tables**

*
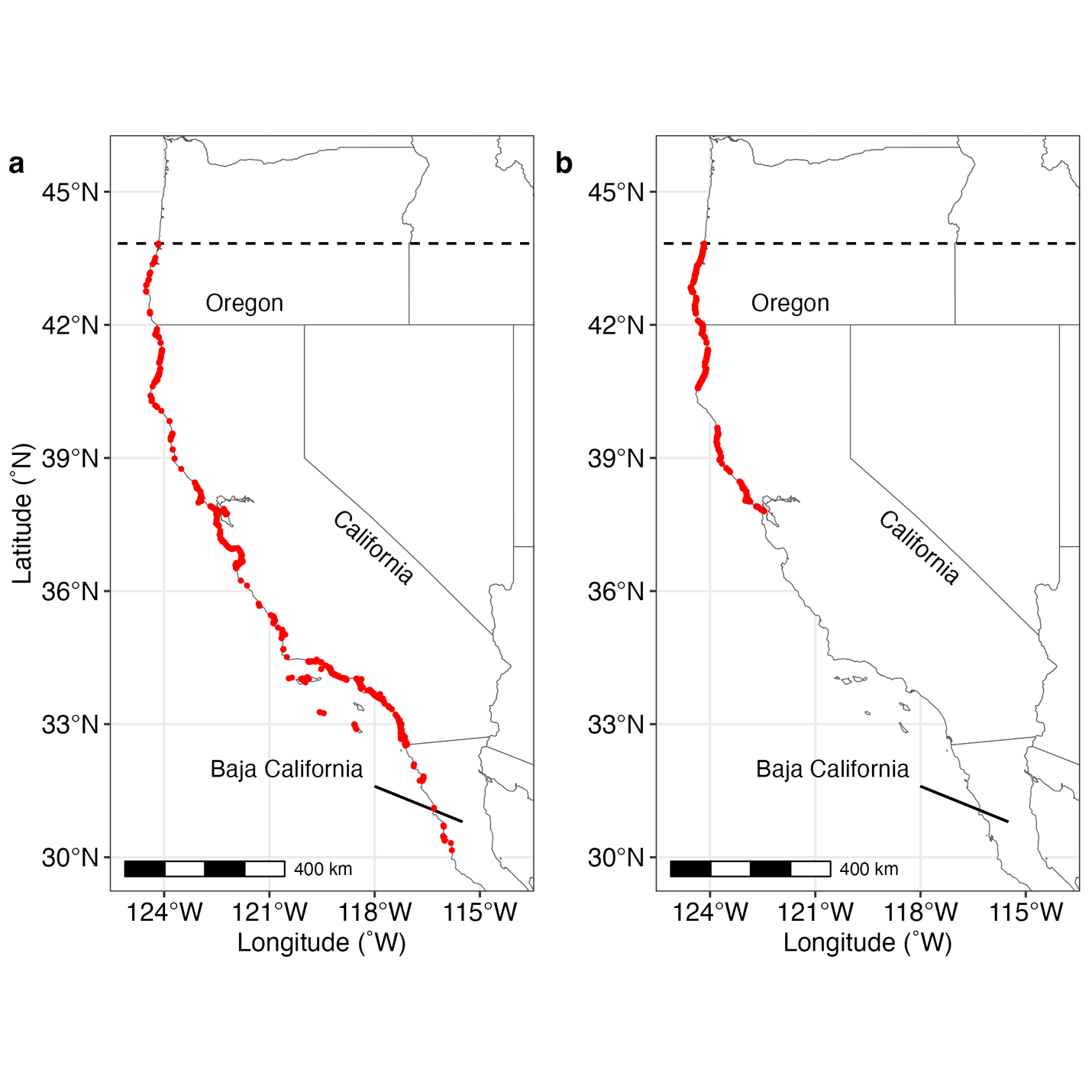
*

*Supporting Figure 1.* (a) Locations of 2631 *Camissoniopsis cheiranthifolia* records on coastal dune habitat from the Global Biodiversity Information Facility (GBIF) accessed January 8, 2024 (GBIF.org 2024). Visualized observations exclude those observed after the revised classification of Onagraceae in 2007 (Wagner & Raven 2007), observations with greater than 1000 m uncertainty, and 12 erroneous inland points. (b) Locations of the 3485 surveyed plots that contained coastal dune habitat. The dashed black lines mark the northern range limit at 43.8˚N. The Lost Coast region in northern California did not contain any survey plots due to the exceptional rarity of coastal dune habitat in the region.


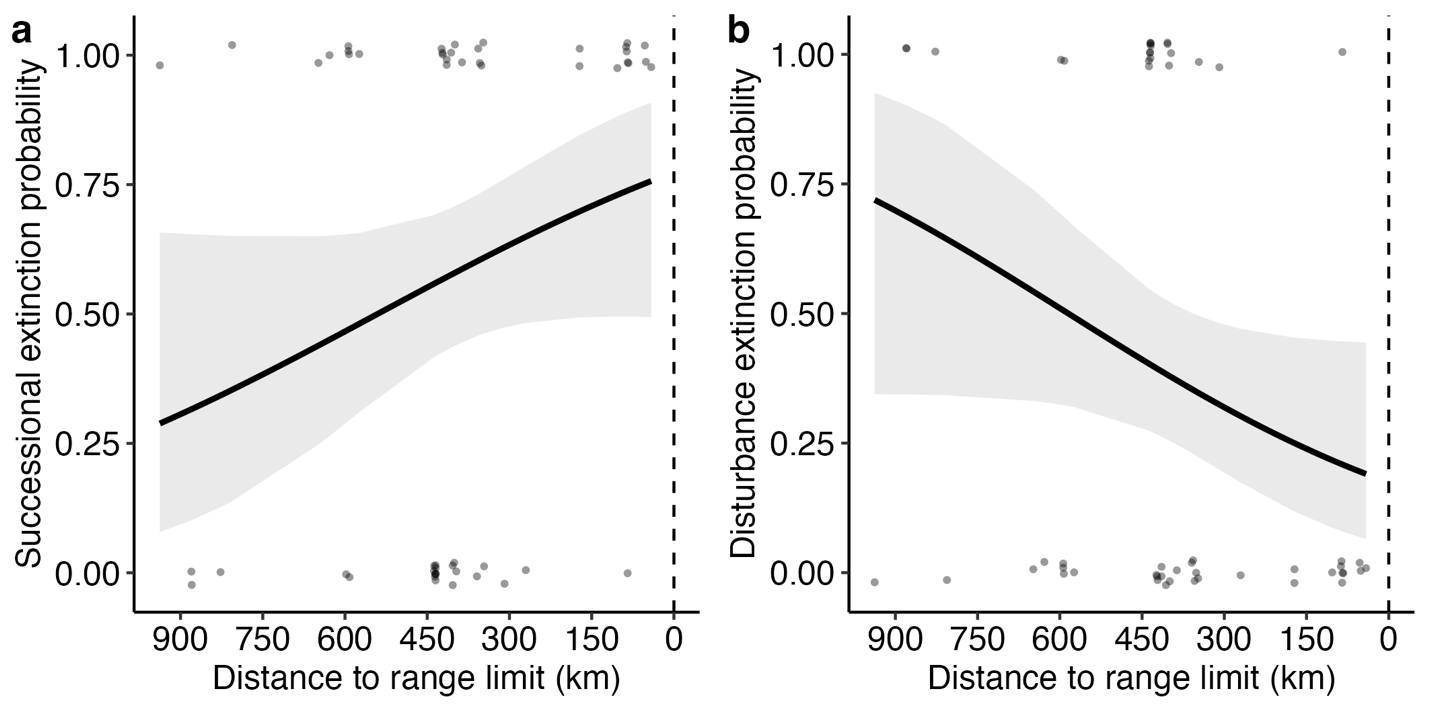
*Supporting Figure 2*. Extinction of *Camissoniopsis cheiranthifolia* from plots caused by (a) succession (i.e., increased vegetation cover) increased non-significantly towards the species’ northern range limit (*n* = 54, *b* = –2.28 x 10^–3^ log-odds, χ^2^ = 2.75, df = 1, *P* = 0.0975), while extinction caused by (b) wind disturbance declined non-significantly towards the range limit (*n* = 54, *b* = 2.67 x 10^–3^ log-odds, χ^2^ = 3.50, df = 1, *P* = 0.0612). A positive coefficient represents an increase in the response variable towards the range core. Grey ribbons represent 95% confidence envelopes. Points are jittered in the vertical axis for clarity.


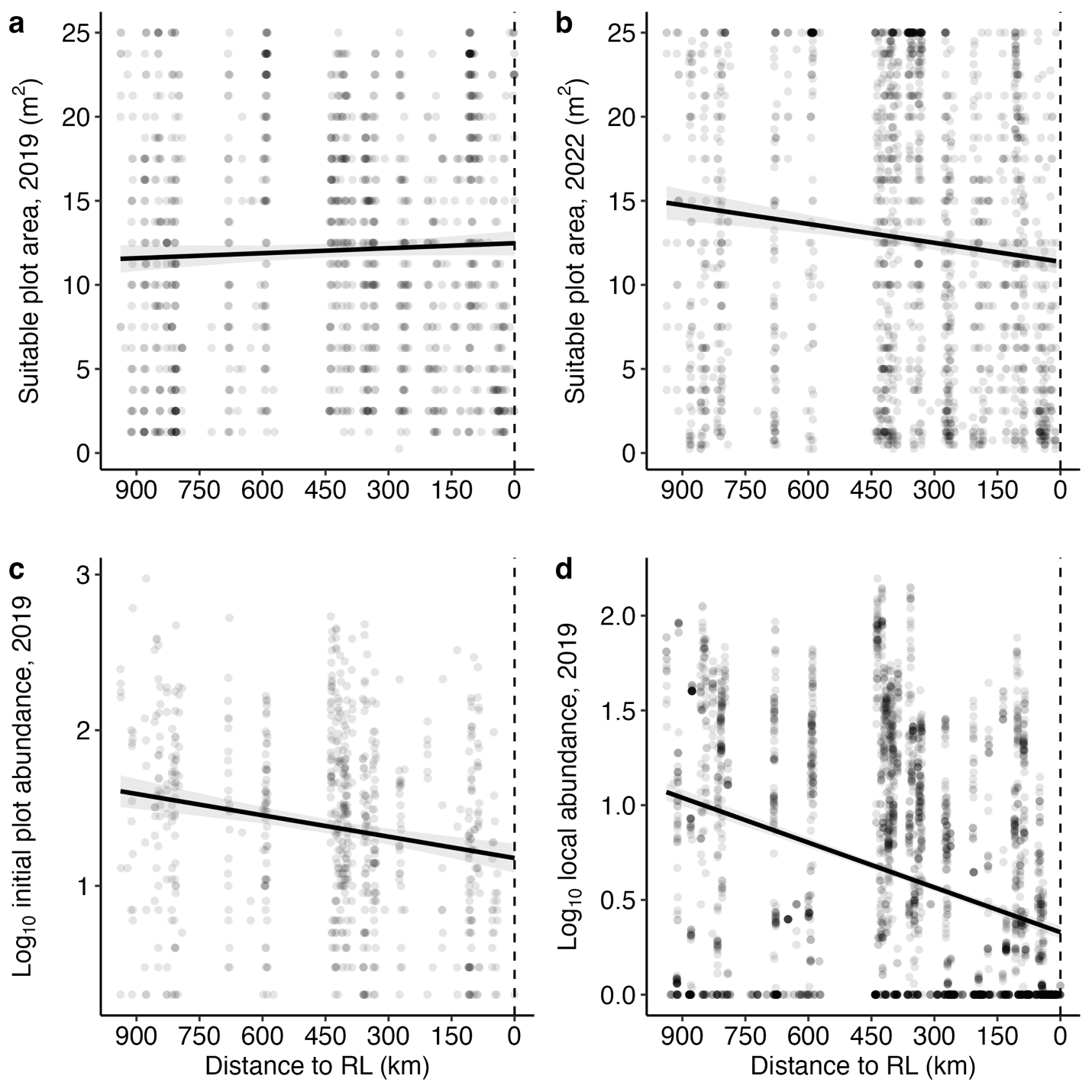


*Supporting Figure 3*. Towards the northern range limit of *Camissoniopsis cheiranthifolia*, (a) 2019 suitable plot area (m^2^) did not vary significantly (Gaussian GLM, *n* = 1264, *b* = –9.90 x 10^–4^, χ^2^ = 2.00, df = 1, *P* = 0.158), (b) 2022 suitable plot area declined significantly (Gaussian GLM, *n* = 1802, *b* = 3.74 x 10^–3^, χ^2^ = 20.90, df = 1, *P* < 0.0001), (c) log_10_-transformed initial plot abundance in 2019 declined significantly (Gaussian GLM, *n* = 686, *b* = 4.58 x 10^–4^, χ^2^ = 24.39, df = 1, *P* < 0.0001), and (d) log_10_-transformed local abundance (average number of *C. cheiranthifolia* in all plots within 500 m of the focal plot) in 2019 declined significantly (Gaussian GLM, *n* = 3473, *b* = 7.89 x 10^–4^, χ^2^ = 460.06, df = 1, *P* < 0.0001). Positive coefficients indicate an increase in the response variable southward to the range core. Grey ribbons show 95% confidence envelopes.

**Table S1:** Summaries for colonization and extinction models that include either distance to the range limit as the sole predictor or distance to the range limit as well as the significant patch/population parameters (Fig. 2). Changes to the slopes (in log-odds units) of the latitude predictor after the inclusion of patch/population parameters indicate that the included parameters explain geographic variation in colonization/extinction.

| Response variable | Model | Predictor | *b* | df | χ^2^ | *P*-value |
| --- | --- | --- | --- | --- | --- | --- |
| Colonization | Only distance to RL | Distance to range limit (km) | 0.001 | 1 | 13.46 | < 0.001 |
|  | Distance to RL + significant patch/  population terms | Distance to range limit (km) | 0.00015 | 1 | 0.19 | 0.66 |
|  |  | Log_10_ local abundance | 0.99 | 1 | 51.16 | < 0.001 |
|  |  | Suitable plot area (m^2^) | 0.079 | 1 | 75.35 | < 0.001 |
| Extinction | Only distance to RL | Distance to range limit (km) | –0.00034 | 1 | 0.83 | 0.36 |
|  | Distance to RL + significant patch/  population terms | Distance to range limit (km) | 0.000033 | 1 | 0.0062 | 0.94 |
|  |  | Log_10_ initial abundance | –0.88 | 1 | 24.58 | < 0.001 |
|  |  | Suitable plot area (m^2^) | –0.021 | 1 | 2.41 | 0.12 |

**Supporting Information S2 – Survey Design & Spatial Data Processing**

Using Google Earth Pro software, we captured aerial images of coastal habitat, stretching across 938 km of coastline between San Francisco, CA, north to Dunes City, OR, encompassing the northern portion of *Camissoniopsis cheiranthifolia*’s range. Using geospatial software ArcMap (v. 10.8) and these aerial images, we identified areas of coastal dune habitat then generated 5,418 sampling points randomly located within this putative coastal dune habitat across the entire study region.

Most predictions we tested analyze change towards and beyond the *C. cheiranthifolia*’s range limit. Distance to the range limit could be measured “as the crow flies” but a measure that is more relevant to the dispersal of the species through coastal dune habitat would be the distance towards the northern range edge along the coastline (Dungey 2021). We obtained aerial Google Earth Pro images taken at a 5 km altitude and sufficient resolution to distinguish coastal dune habitat from adjacent beach habitat and stabilized habitat further upland. Each image was ~3.5 km long latitudinally and at least 1 km wide longitudinally, and collectively included all coastal habitat within the study area. Images were downloaded as .jpg files at maximum resolution. We used the Iso Cluster Unsupervised Classification tool in the geospatial software ArcMap (v. 10.8) to separate RGB image pixels into either shoreline or water thereby defining the coastline in each image (Farris *et al.* 2019; Konko *et al.* 2020). We then used the Create Features tool to convert the extracted coastline pixels into polyline shapefiles. We manually adjusted shapefiles using the Smooth Boundaries and Edit Features tools to outline the entire coast of the study region. We merged the individual shapefiles generated from each image into a single shapefile (hereafter the ‘coastal reference line’) that outlines the entire coast of the study area.

Within the identified coastal dune habitat, we randomly distributed 5,418 sampling locations. We first converted all Google Earth Pro images that included coastal dune habitat into .mapx files for spatial analysis with ArcMap. We georeferenced each image based on ≥ 10 control points using the Georeferencing tool based on the World Geodetic System 1984 (Kumar 1988). This yielded accurate latitude/longitude coordinates for every pixel within a given map. For each map, we outlined the areas of potentially suitable habitat (PSH) based on ecological criteria developed by previous range-wide surveys of *C. cheiranthifolia* (Table S2) (Samis & Eckert 2007, 2009). We extracted areas of PSH by using the Extract by Polygon tool within the ArcMap Spatial Analyst toolset to isolate the polygons of PSH. The resulting polygons were conjoined into a single polygon shapefile using the Dissolve tool.

Within each PSH shapefile, we overlaid a grid of 10 m^2^ cells using the Create Fishnet tool and used the Create Random Points tool to generate coordinates for sampling points within randomly selected cells. The number of sampling points within each PSH shapefile was adjusted such that ~15 points were placed per km of coastline. Supplementary analyses confirmed there was no systematic geographic variation in the density of sampling points towards the range limit given the amount of PSH identified, as well as in the distance between points (Dungey 2021). We recorded the latitude and longitude of each sampling point in decimal-degrees to four decimal places and calculated the distance of each point to the species’ northern range limit (the northernmost observed plot containing *C. cheiranthifolia* in either survey year, 43.83328˚N) as the shortest distance from the point to the coastline plus the distance along the coastline to the range limit. The generated sampling point coordinates were uploaded to handheld Garmin GPSmap 60Cx using the Garmin BaseCamp geospatial software (v. 4.8.6).

**Table S2**. Ecological criteria used to identify suitable habitat for *Camissoniopsis cheiranthifolia* at both coarse and fine spatial scales. The coarse scale criteria were used to identify and extract potentially suitable habitat from coastal dune imagery captured in Google Earth Pro (GEP). The fine scale criteria were used to identify and measure the amount of suitable habitat within randomly located 5m x 5m sampling plots during field surveys. Criteria were determined from previous range-wide surveys of *C. cheiranthifolia* (Samis & Eckert 2007, 2009).

| Habitat variable | Criteria for determining suitable *C. cheiranthifolia* habitat | |
| --- | --- | --- |
|  | Coarse scale (aerial imagery) | Fine scale (sampling plots) |
| Location | - Either directly along coastline, or inland but within <1km of coast - Dune system accessible to coastline, unimpeded by other landscape types (e.g., forest, estuary, rock beach, human development) - Presence of identifiable dune characteristics (beach front with tide line, foredune and/or sand hummocks, deflation plain, sand flats, etc.) | - Areas of sand ranging from the leading foredune slope, inland into the deflation plain - In the absence of foredune, areas of sand hummock formation, well above tideline - Outside areas within reach of tidal, estuary overflow - Outside areas with visible signs of frequent human disturbance (e.g., trails, dune buggy tracks, development) |
| Substrate | - Predominantly sand substrate; light to medium beige colours - Areas of sand visible amongst patches of plant cover - Substrate inland of high tide, no evidence of flooding (darker colouration) | - Substrate consists of coarse silt to medium sand - Semi-stabilized sand, determined from presence/absence of native species and/or abiotic features - Low organic content, determined by substrate colour (light beige) |
| Elevation and slope | - Elevation and slope not discernible from GEP images | - Elevation < 50 m - Foredune and back-dune slopes < 60˚ |
| Disturbance | - Areas show little to no disturbance from tidal flooding, anthropogenic disturbance (i.e., darker substrate colour, tide lines) - Presence of natural debris among sand patches (i.e., driftwood) - Dune system largely free of anthropogenic development (i.e., housing, industrial areas, parking lots, etc.) | - Semi-disturbed sand, stabilized by abiotic debris and native sand stabilizer plant species - Sand is not fully malleable or fully compacted, should sink slightly underfoot - Protection from intense wind disturbance |
| Exposure | - Foredune, deflation plain easily visible among vegetation cover - Patches of open sand are not blocked from overhead view, clearly visible & interspersed among unsuitable habitat cover, abiotic debris, and anthropogenic cover | - Sand exposed enough to experience periodic disturbance; unprotected by large abiotic cover (large debris, rocks, etc.) - May occur in more exposed sand sheets if 1) large foredune is present, or 2) within deflation plain |
| Plant community | - Sections of green colour distributed across foredune/deflation plain - Areas of sand visible among plant cover; avoid areas of largely homogenous green vegetation | - Presence of native sand stabilizers (e.g., *Elymus mollis*, *Ambrosia chammisonis*) and semi-stabilizing endemics (e.g., *Erigeron glaucus*, *Potentilla pacifica*) - Presence of invasive sand stabilizers (e.g., *Ammophila arenaria*, *Carpobrotus* spp.) - Sand patches not dominated by large densely packed woody dune species (e.g., *Baccharis pilularis*, *Cytisus scoparius*, *Ulex europaeus*, *Pinus contorta*, *Picea sitchensis*) |

**Supporting Information S3 – Evaluating Relocation Error**

We used two approaches to evaluate the error in calculating colonization and extinction associated with uncertainty in relocating the centre of each plot in 2022. First, we staked many locations in dune habitat and then relocated them the following day using hand-held GPS devices. Relocation error was never more than ~1.5 m. Second, for all plots we recorded occupancy and suitability in each of the four 5m x 5m areas adjacent to and abutting each side of the central plot (hereafter ‘adjacent plots’) in 2022. Among 661 occupied focal plots, 76% of adjacent plots were also occupied, while 97% of plots adjacent to 2317 vacant plots were also vacant. Among 1690 suitable plots, 89% of adjacent plots were also suitable, while 95% of plots adjacent to 1295 unsuitable plots were also unsuitable. Thus, adjacent plots tend to have very similar occupancy and suitability status as focal plots, suggesting that GPS-induced measurement error would not introduce much error into calculating colonization and extinction. Moreover, there is no reason to expect that this source of error would vary geographically.

**References**

Dungey, M.P. (2021). A broad scale investigation of dispersal constraints on the northern range limit of a Pacific coastal dune plant. Master’s Thesis, Queen’s University.

Farris, A.S., Defne, Z. & Ganju, N.K. (2019). Identifying salt marsh shorelines from remotely sensed elevation data and imagery. *Remote Sens. (Basel)*, 11, 1795.

GBIF.org. (2024). GBIF Occurrence Download.

Konko, Y., Okhimambe, A., Nimon, P., Asaana, J., Rudant, J.P. & Kokou, K. (2020). Coastline change modelling induced by climate change using geospatial techniques in Togo (west Africa). *Adv. Remote Sens.*, 09, 85–100.

Kumar, M. (1988). World geodetic system 1984: A modern and accurate global reference frame. *Mar. Geod.*, 12, 117–126.

Samis, K.E. & Eckert, C.G. (2007). Testing the abundant center model using range-wide demographic surveys of two coastal dune plants. *Ecology*, 88, 1747–1758.

Samis, K.E. & Eckert, C.G. (2009). Ecological correlates of fitness across the northern geographic range limit of a Pacific Coast dune plant. *Ecology*, 90, 3051–3061.

Wagner, W.L. & Raven, P.H. (2007). Revised classification of the Onagraceae. *Systematic Botany Monographs*.
